# Government-Nongovernmental Organization (NGO) Collaboration in Macao’s COVID-19 Vaccine Promotion: Social Media Case Study

**DOI:** 10.2196/51113

**Published:** 2024-03-19

**Authors:** Xuechang Xian, Rostam J Neuwirth, Angela Chang

**Affiliations:** 1 Department of Publicity Zhaoqing University Zhaoqing China; 2 Department of Communication University of Macau Macao SAR China; 3 Department of Global Legal Studies University of Macau Macao SAR China

**Keywords:** COVID-19, government, vaccine, automated content analysis, Granger causality test, network agenda setting, QAP, social media

## Abstract

**Background:**

The COVID-19 pandemic triggered unprecedented global vaccination efforts, with social media being a popular tool for vaccine promotion.

**Objective:**

This study probes into Macao’s COVID-19 vaccine communication dynamics, with a focus on the multifaceted impacts of government agendas on social media.

**Methods:**

We scrutinized 22,986 vaccine-related Facebook posts from January 2020 to August 2022 in Macao. Using automated content analysis and advanced statistical methods, we unveiled intricate agenda dynamics between government and nongovernment entities.

**Results:**

“Vaccine importance” and “COVID-19 risk” were the most prominent topics co-occurring in the overall vaccine communication. The government tended to emphasize “COVID-19 risk” and “vaccine effectiveness,” while regular users prioritized vaccine safety and distribution, indicating a discrepancy in these agendas. Nonetheless, the government has limited impact on regular users in the aspects of vaccine importance, accessibility, affordability, and trust in experts. The agendas of government and nongovernment users intertwined, illustrating complex interactions.

**Conclusions:**

This study reveals the influence of government agendas on public discourse, impacting environmental awareness, public health education, and the social dynamics of inclusive communication during health crises. Inclusive strategies, accommodating public concerns, and involving diverse stakeholders are paramount for effective social media communication during health crises.

## Introduction

As of December 2022, the global COVID-19 pandemic had resulted in 669 million confirmed cases and 6.8 million deaths [1]. Environmental factors were a key determinant significantly influencing the pandemic [2], through airborne viral infectivity impacted by air pollution and seasonality effects [3,4].

Vaccination was crucial to contain the spread of virus [5], although complex factors such as the Peltzman effect, emerging viral variants, and socioeconomic conditions also affected pandemic diffusion [6]. Determining an optimal level of vaccination is complex and multifaceted, requiring a balance to avoid undermining democratic values and triggering larger socioeconomic problems than the pandemic [7,8]. Nonetheless, the willingness to vaccinate hinges on various factors, including safety concerns, sociodemographic characteristics, and individual behaviors and attitudes [9,10]. Other determinants including lack of knowledge, government distrust, skepticism about vaccine development, efficacy concerns, exposure experience, coronaphobia, and workplace mandates also predict vaccine uptake [11-13]. As social media becomes increasingly significant for public communication, social media adaptivity, information availability, and health care infrastructure capabilities are also influential for vaccination decisions [14].

Vaccine communication plays a vital role in addressing public concerns, building trust, and encouraging vaccine uptake. Specifically, effective strategies including trusted sources, health provider guidance, a reasonable quantity of information, cultural tailoring, information contextualization, and cultural sensitivity have the potential to significantly increase vaccination intent [15-17]. Despite the notable antagonism in the discourse surrounding immunization on social media [18], it is worth noting that social media campaigns initiated by health organizations have proven to be effective in increasing public awareness about vaccination [19].

Governance mechanisms are another crucial factor for expediting vaccine distribution and mitigating pandemic-related socioeconomic effects [20]. Evidence has shown that clear, consistent, and transparent communication from governmental bodies engendered higher levels of public compliance and trust [21,22]. Given the major impact of the pandemic on public health and society, involvement of the government in vaccine communication becomes a vital research area.

Governments worldwide have adopted diverse approaches to encourage COVID-19 vaccination. For instance, the New Zealand government promoted vaccination among young people by highlighting community factors such as “protecting others” and “striving for herd immunity” [23]. By promoting the scientific notion that there are more advantages than disadvantages to COVID-19 vaccination, the Chinese government has strengthened risk communication to increase the public's awareness of the benefits of vaccines [24]. Although COVID-19 vaccine communication has received increasing attention, particularly from the research community, scientific evidence focusing specifically on low-risk regions, such as Macao, is scarce. This suggests that the existing literature does not sufficiently reflect the concerns of the Macao population as related to COVID-19 vaccination. As one of the world’s most densely populated cities, Macao has maintained a record of relatively low risk of infection and high coverage of COVID-19 vaccines [25]. Throughout the pandemic before June 2022, Macau had only recorded 17 confirmed cases of local infection (with a rate of 2.5 cases per 100,000 population) with no fatalities. By June 19, 2022, the vaccine coverage rate within the entire population in Macao was 85.6% for at least 2 doses and 40.5% for 3 doses [26]. The low prevalence of COVID-19 is believed to be the result of the close connection between Macao and mainland China. Since the outbreak of the pandemic, Macao has implemented anti-epidemic measures following the “dynamic zero-COVID-19 policy” established by mainland China, with some adaptations based on local socioeconomic circumstances [27]. Given the close link between these entities, it is important to understand how the Macao Government communicated with citizens to drive their demand for vaccinations and the impact of this communication. Researchers have long investigated how governments develop policy agendas and whether a policy agenda is led by the government or the public [28]. However, literature on the role of the government in public health agenda setting, specifically related to vaccine promotion in the COVID-19 context, is limited.

The primary goal of this study was to reveal the patterns of vaccine communication on social media during the COVID-19 pandemic as well as the role of the government in advancing vaccination through a case study of Macao, the special administrative region of China. By conducting this research, we aimed to contribute to the existing knowledge on vaccine communication and provide implications for policymakers to improve health promotion communication strategies for preparedness against future pandemics.

The theory of agenda setting suggests that the media has the ability to influence the public agenda by making a specific issue prevalent and salient [29]. Agenda setting is a competition among issue proponents to gain the attention of media professionals, the public, and policy elites [30]. Recently, research about agenda setting has been extended by incorporating the concept of social networks and the associative network of memory, which has been proposed by Guo [31] as the network agenda setting model (NAS). The NAS underlines the associations between topics or attributes presented in the agenda: The more frequently 2 attributes are correlated in news coverage, the more likely the public will perceive them to be interrelated [32].

The NAS can be used to identify the interconnections between public, media, organizational, and government topics on social media. For instance, a study conducted by Chen et al [33] utilized the NAS to investigate the correlation between individual users and organizational accounts on Weibo in terms of their focus on nationalist concerns. The NAS emphasizes the relationship between topics or attributes in constructed agendas. Hou et al [34] analyzed posts mentioning COVID-19 vaccines on Twitter and found that topics related to COVID-19 vaccines can be divided into the following 9 categories: (1) vaccine importance, (2) vaccine effectiveness, (3) vaccine safety, (4) trust in governments, (5) trust in experts, (6) COVID-19 risk, (7) vaccine accessibility, (8) vaccine distribution, (9) vaccine affordability. Additionally, recent studies examined the concerns of all users, including parents, regarding COVID-19 vaccines (eg, [35]). However, these studies did not distinguish between regular accounts (ie, ordinary individual users), government accounts, organization accounts, and media accounts. This distinction is important to understand the nuances of vaccine promotion engaged by different entities. Governments, for instance, influence public discourse through policymaking [24,28], whereas organizations play a significant role in agenda setting via funding, lobbying, and advertising activities [36]. The public, media, and government may construct different associations among topics in their respective agendas and impact each other. Our research questions (RQ) thus ask the following:

- RQ1: What are the most prevalent agenda attributes emphasized in the communication of vaccination on Facebook during the COVID-19 outbreak in Macao?
- RQ2: How do the attributes interact in the vaccine agendas of governmental and nongovernmental entities?

- RQ3: What are the associations between the vaccine agenda networks constructed by government and nongovernment users?
- RQ4: How do government and nongovernment users impact each other’s vaccine agenda on Facebook?

## Methods

### Sample and Data

This study retrieved data relevant to COVID-19 vaccines in Macao from January 1, 2020, when the SARS-CoV-2 virus was initially detected in China, to August 31, 2022, when the number of newly reported cases had sharply declined [1]. Facebook was selected as the main source of data to analyze the dynamics of vaccine communication in Macao. Being one of the most widely used social media platforms globally, Facebook accounts for a more dominant market share (65.05%) than other sources (eg, Pinterest: 11.47%; Twitter: 10.54%) in Macao [37,38]. The widespread usage of Facebook suggests that it has a significant impact on the population’s perceptions, attitudes, and behaviors, making it an essential platform to study to understand the public agenda. In addition, Facebook’s archival nature allows for tracking of the evolution of vaccine-related discussions over time, capturing the core dynamics of vaccine communication online.

A combination of the keywords “COVID-19” and “vaccine” as well as their synonyms (ie, 29 synonyms of COVID-19–related terms and 10 synonyms of vaccine-related terms) in Chinese were used to detect and collect relevant posts (see Multimedia Appendix 1). Information was also compiled on the various labels given to users on Facebook, such as labels of government, media, and organization accounts. Following the collection of raw data from Facebook, data screening was performed to remove duplicate and irrelevant posts. The preprocessing of data including the removal of stop words (eg, “an,” “the,” “etc.,” punctuation, symbols, and numbers) and word segmentation was implemented using the DivoMiner platform.

### Ethics Approval

This research strictly adheres to ethical guidelines by ensuring complete anonymity and de-identification of all data sources. To preserve the confidentiality and privacy of all sources involved, no identifiable information about individual users, their IDs, or direct, non-paraphrased posts are included in the main manuscript or any supplementary materials.

### Clarification

All identifiers in the data set (eg, names of the senders) were removed and replaced with a code to mask the information about each sender, ensuring the anonymization of our data. Data were only collected from publicly available posts that were returned based on the structured keyword search criteria.

### Measures of Variables

This study investigated the dynamics of agenda setting between government and nongovernment users on Facebook. To achieve this, we categorized users into the following different categories, drawing from prior research [39,40]: (1) media, (2) civil organizations, (3) regular users, (4) government.

The media functions as information gatekeepers and holds potential influence over people’s decision-making [29,32]. To account for significant differences in content, news culture, and viewpoints, the media category in this study was further divided into professional media and alternative media for a thorough investigation [41]. Professional media includes those traditional mass media outlets responsible for information dissemination and public awareness, such as newspapers, radio, and television, while alternative media includes independent and electronic media, which is in contrast to mainstream mass media. By referencing relevant media research [42], this study annotated professional media accounts, alongside alternative media accounts.

Civil organizations, also called civil society organizations, include those organizations or associations that are established by individuals or groups with a common purpose or interest and operate in the community, differing from the government and corporations. Civil organizations work alongside the government and other stakeholders to contribute to public discourse, policy development, and social change [43].

Regular users were defined in this study as individuals who interact with Facebook on a personal basis, without representing any official capacity, media, or organizations. Therefore, regular users can be considered as representatives of the public in this study.

The government in this study was defined as all authorities. We did not categorize the specific levels, instead treating all government authorities as a single entity, to gain a clear understanding of the overall position of the Macao Government in vaccine communication. This was also a practice adopted by previous research (eg, [44]).

The classification of Facebook users into 5 distinct categories was conducted based on the information gathered from users’ short biographical profiles and the user identity labels provided by Facebook. We assigned 2 coders to classify the users contributing relevant posts. Any confusion that might have occurred during classification was resolved through discussion. This approach allowed for the categorization of users into specific groups, enabling a systematic analysis of user communication and interactions within the Facebook platform [44].

To investigate the dynamics of vaccine communication, 9 predefined categories that indicate elements influencing vaccine acceptance were established based on a coding framework adapted from prior studies (eg, [34,45,46]). These categories included the following topics: importance of vaccines, effectiveness of vaccines, safety of vaccines, trust in governments, trust in experts, risk of the COVID-19 pandemic, and vaccine convenience (ie, accessibility, distribution, and affordability). Details of the coding categories are shown in Multimedia Appendix 2.

### Data Analysis Procedures

#### Automated Content Analysis

In this study, an automated content analysis method was used to identify and categorize posts into the predefined categories. Each post could belong to one or more categories or none at all. The effectiveness of automated coding depends on the design of the keywords. To develop accurate keywords, this study followed the approach outlined by Chang et al [37] using the Word2vec word embedding toolkit from the Python 3.7.4 Gensim module [47]. Word2vec, a word embedding technique powered by neural networks, allows the identification of words with similar meanings by analyzing word associations in a large text corpus [48]. Due to the intricacies of the Chinese language, the synonyms suggested by Word2vec were further checked by assessing their relevance to the context. On this basis, the Chinese thesaurus and relevant literature [49] were further consulted for the inclusion of additional synonyms. The list of keywords for machine coding can be found in Multimedia Appendix 3.

DivoMiner, a text mining and automated content analysis platform driven by machine learning algorithms, was used to facilitate the automated content coding task. This platform integrates automated content analysis with traditional content analysis methods and has been widely utilized in health and communication studies [37,50,51]. Following automated coding, manual verification was conducted to ensure the accuracy and reliability of the machine-generated outcomes. To achieve this, 2 coders, both native Cantonese speakers, were recruited and underwent 36 hours of training to independently code 300 messages. Each variable was coded as either present or absent. Discrepancies between the coders were resolved through discussions, with the author intervening only when consensus could not be reached between the coders. The overall intercoder reliability, measured using Krippendorff alpha, demonstrated satisfactory levels across all examined variables, with coefficients ranging from .77 to .82. The consistency between machine coding and manual coding reached an acceptable level, with an average score of 74%. This score aligns with previous studies, in which a threshold value of 70% was considered rational [49-51].

#### Statistical Analysis

The conventional statistical analysis in this study involved the use of SPSS (version 23; IBM Corp) for analysis. Categorical variables were summarized using counts and percentages. The chi-square test of independence was used, and post hoc comparisons with Bonferroni corrections were further implemented to precisely identify the specific significant differences between user categories and vaccine-related topics and avoid the likelihood of generating false-positive outcomes (type I errors).

#### Co-Occurrence Network Analysis

Co-occurrence matrices, which represent the strength of ties between 2 topics engaged by different users, were generated as dyadic data sets. Based on the co-occurrence data, this study established undirected and weighted topic co-occurrence networks. Each network represents the co-occurrence relations of the attributes of a certain user category. To clarify, if a particular category of user mentions topic “i“ and topic “j,” a band will link “i” and “j.” The width of the band indicates the frequencies of the pair of topics discussed by a user type [52,53]. For example, in the professional media user category’s topic co-occurrence network, if a professional media news report mentions the topics of “vaccine importance” and “vaccine effectiveness” together, the topics will be linked in the network by a band. The more frequently these topics co-occur, the thicker the band becomes. The visualization of topic co-occurrence is presented in a chord diagram by Echarts (The Apache Software Foundation), as indicated by Wang et al [52].

#### Quadratic Assignment Procedure for Network Analysis

In this study, the quadratic assignment procedure (QAP) method was applied to understand the correlation between the Macao Government’s agenda network and that of other Facebook users, via analysis of the co-occurrence matrices. QAP is a common method in social network or agenda network studies [40,54]. QAP correlation analysis can be used to assess the correlation between 2 matrices with the Pearson correlation coefficient, while QAP regression analysis can determine whether an explanatory variable can predict an outcome variable when the 2 matrices are significantly correlated [55]. In this study, the QAP method used UCINET 6.730 to test whether the Macao Government’s vaccine agenda network has impacted that of nongovernment Facebook users, particularly regular type users, during the COVID-19 pandemic.

#### Vector Autoregression Modeling

The vector autoregression (VAR) approach was used to examine the dynamic of agenda attributes between government and nongovernment users. This approach evaluates the effect of an observed variable by considering its lagged effect in the earlier period and that of other predictors in previous time points, without presuming the associations between the variables [56]. The VAR modeling technique is widely used in the economic field and, in recent years, has been increasingly applied in research on health science, sociology, neuroimaging, and meteorology (eg, [54,57-59]).

VAR modeling is ideal for measuring the dynamic performance response and interaction between performance and marketing communication variables. A study applied VAR models to construct the dynamic response relationship between news stories and public attention using a combination of survey and news content ranging from 2009 to 2013 [60]. The VAR models captured the dynamic feedback system and gave estimates for the short-term effects of TV news coverage on public perception by demonstrating a unidirectional process wherein changes in news salience led to significant changes in public salience. In addition, VAR models have also been used to investigate the dynamic mapping relationship between the diffusion of political messages and emotional expression in public messages during the COVID-19 pandemic [61]. The increased diffusion of political messages positively predicted changes in emotional expression among citizens, and the VAR model was able to explain the interdependencies among variables based on the lag values of multiple time series. Overall, the VAR model proves to be an insightful tool for analyzing complex relationships in communication studies, providing insights into the short-term and long-term effects of various factors on outcomes of interest. Hence, using the VAR technique allows the exploration of temporal dynamics and associations between different agenda attributes in this study. For example, the approach enables a better understanding of whether the agenda attributes propagated by the government (AG) at time (t-n) impacts the agenda attributes of nongovernment users (AN) including professional media, alternative media, civil organizations, and regular users. The VAR model was generated as follows:



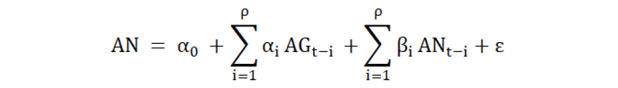



Within this model, α_i_ and β_i_ are the estimated coefficients, ρ represents the optimal number of lags for the model, and ε indicates the error term. AG_t-i_ and AN_t-i_ represent the respective variable at the earlier periods. For instance, AG_t-1_ indicates the first lag of AG. The lag length for the VAR model was selected as per the Akaike information criterion. The augmented Dickey-Fuller test was applied to examine the stationarity of the time series. For nonstationary series, differencing at the first or higher level was performed to achieve stationarity [62]. When both time series were stationary at the same level, this study proceeded with the Johansen maximum eigenvalue and trace tests based on the estimation of VAR models to determine whether the time series were cointegrated and suitable for Granger causality tests. Granger causality posits that causes lead to effects and happen before their effects [40]. In this sense, using prior values of a time series can statistically forecast the future status of another time series.

In this study, the Granger causality test was used to provide greater insight into the statistical causal relationship between the government’s agenda and the nongovernment users’ agenda. To estimate VAR models and enable Granger causality tests, this study transformed the collected data in the form of time series by dividing the data into 32 monthly periods (from January 2020 to August 2022), and each monthly period was treated as an independent unit for analysis. EViews 12 software was used for statistical analysis.

## Results

### Results of Content Analysis

This research initially collected a sample of 24,089 Facebook posts with relevance to COVID-19 vaccines. Data screening was further performed on the sample to remove duplicated, irrelevant, and unclear messages, resulting in 23,577 unique and relevant posts. Finally, the results of machine coding presented a total of 22,986 posts that include the examined vaccine topics.

In answering RQ1, we calculated the frequency of the vaccine topics and found that the majority of posts in the sample related to the importance of COVID-19 vaccination (7358/22,986, 32.01%), followed by posts that indicated the high risk of contracting COVID-19 (6877/22,986, 29.92%) and highlighted trust in experts (4320/22,986, 18.79%). In addition, a considerable number of posts mentioned vaccine effectiveness (4163/22,986, 18.11%), safety (3358/22,986, 14.61%), accessibility (2683/22,986, 11.67%), distribution (2492/22,986, 10.84%), and affordability (1685/22,986, 7.33%), while posts related to trust in government were less frequent (1593/22,986, 6.93%). In addition, in the overall vaccine-related discussion, nongovernment users comprised a substantial majority of the posts, at 76.85% (17,665/22,986). When examining the nongovernment user segment at a more granular level, professional media accounted for a significant proportion of the posts, at 33.87% (7555/22,986), followed by alternative media, at 12.24% (2814/22,986); civil organizations, at 3.99% (918/22,986); and regular users, at 27.74% (6377/22,986). The topics associated with vaccine agenda attributes by government and nongovernment users are shown in Table 1.

The chi-square test indicated that the distributions of vaccine-related topics were significantly different across the user categories (χ^2^_32_=1579.469, *P*<.001). The outcomes of the post hoc comparisons suggested that the government was more concerned with topics of vaccine effectiveness (1003/5322, 18.85%; *P*<.001), COVID-19 risk (1805/5322, 33.92%; *P*<.001), vaccine accessibility (1010/5322, 18.98%; *P*<.001), and vaccine affordability (605/5322, 11.37%; *P*<.001), while discussion of vaccine safety (393/5322, 7.38%; *P*<.001), government trust (133/5322; 2.5%, *P*<.001), expert trust (518/5322, 9.73%; *P*<.001), and vaccine distribution (341/5322, 6.41%; *P*<.001) occurred to a less extent than for other users. In comparison, professional media contributed more to the topics of government trust (752/7555, 9.95%; *P*<.001) and expert trust (1895/7555, 25.08%; *P*<.001). Alternative media, however, were less inclined to discuss vaccine affordability (128/2814, 4.55%; *P*<.001) than other categories of users. Regular users were primarily concerned about vaccine safety (1092/6377, 17.12%; *P*<.001) and vaccine distribution (724/6377, 11.35%; *P*<.001) and were less concerned about vaccine effectiveness (937/6377, 14.69%; *P*<.001), COVID-19 risk (1529/6377, 23.98%; *P*<.001), and vaccine accessibility (416/6377, 6.52%; *P*<.001) than other users. The outcomes of the post hoc tests with details are shown in Multimedia Appendix 4.

**Table 1:** Overview of the vaccine agenda attributes by government and nongovernment users in Macau from January 1, 2020, to August 31, 2022.

Vaccine topic	Government users, n (%)	Nongovernment users, n (%)				Total, n (%)
		Professional media	Alternative media	Civil organizations	Regular users	
All posts	5322 (23.15)	7555 (32.87)	2814 (12.24)	918 (3.99)	6377 (27.74)	22,986 (100)
Importance	1616 (30.36)	2931 (38.80)	697 (24.77)	298 (32.46)	1816 (28.48)	7358 (32.01)
Effectiveness	1003 (18.85)	1638 (21.68)	404 (14.36)	181 (19.72)	937 (14.69)	4163 (18.11)
Safety	393 (7.38)	1374 (18.19)	359 (12.76)	140 (15.25)	1092 (17.12)	3358 (14.61)
Trust in government	133 (2.5)	752 (9.95)	175 (6.22)	40 (4.36)	493 (7.73)	1593 (6.93)
Trust in experts	518 (9.73)	1895 (25.08)	593 (21.07)	154 (16.78)	1160 (18.19)	4320 (18.79)
COVID-19 risk	1805 (33.92)	2651 (35.09)	681 (24.2)	211 (22.98)	1529 (23.98)	6877 (29.92)
Accessibility	1010 (18.98)	981 (12.98)	196 (6.97)	80 (8.71)	416 (6.52)	2683 (11.67)
Distribution	341 (6.41)	1005 (13.3)	309 (10.98)	113 (12.31)	724 (11.35)	2492 (10.84)
Affordability	605 (11.37)	529 (7)	128 (4.55)	53 (5.77)	370 (5.8)	1685 (7.33)

### Trend in Facebook Activities

**Figure 1:**
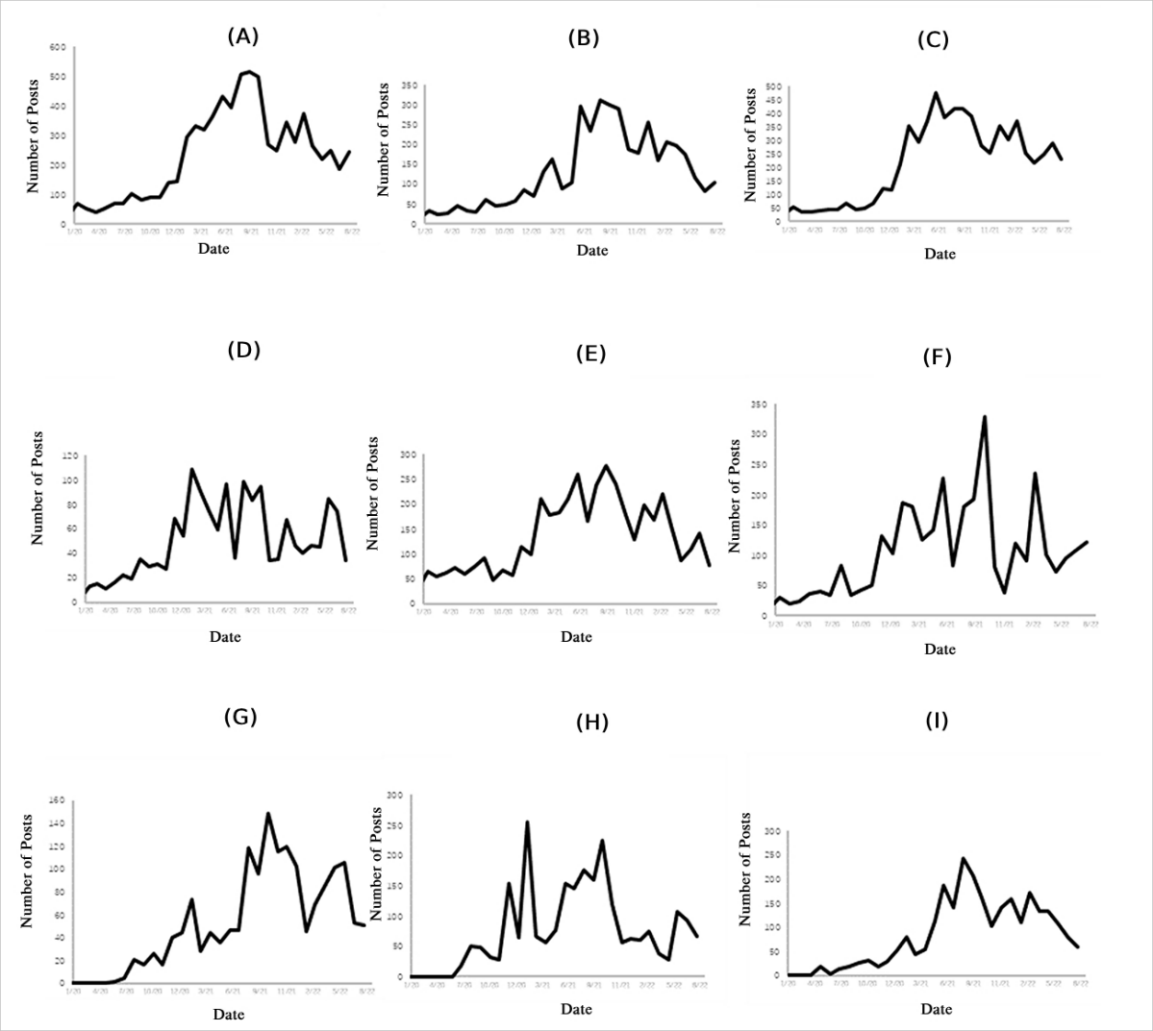
Temporal changes in the vaccine agenda attributes (January 2020–August 2022): (A) vaccine importance, (B) vaccine effectiveness, (C) risk of COVID-19, (D) government trust, (E) expert trust, (F) vaccine safety, (G) vaccine affordability, (H) vaccine distribution, (I) vaccine accessibility.

To reveal the dynamics of different attributes of the vaccine agenda, this study mapped trends of these attributes during the investigated period. All vaccine-relevant content remained at a relatively low volume in 2020 and increased significantly in 2021. The volume of content regarding “vaccine distribution” began to grow at the start of 2021 and showed an observable spike in February of the same year. This was followed by a sharp acceleration in content regarding the high risk of COVID-19 reaching its peak in June 2021. The highest peak in vaccine-relevant content occurred in September 2021 related to the topic of vaccine importance. Between June 2021 and October 2021, the most debate centered around themes relating to COVID-19 vaccines. Overall, variations in the volume of vaccine communication were observed over time. Figure 1 shows the dynamic of vaccine discussion showing the monthly volume of posts.

### Interactions Between Agenda Attributes in Vaccine Communication

**Figure 2:**
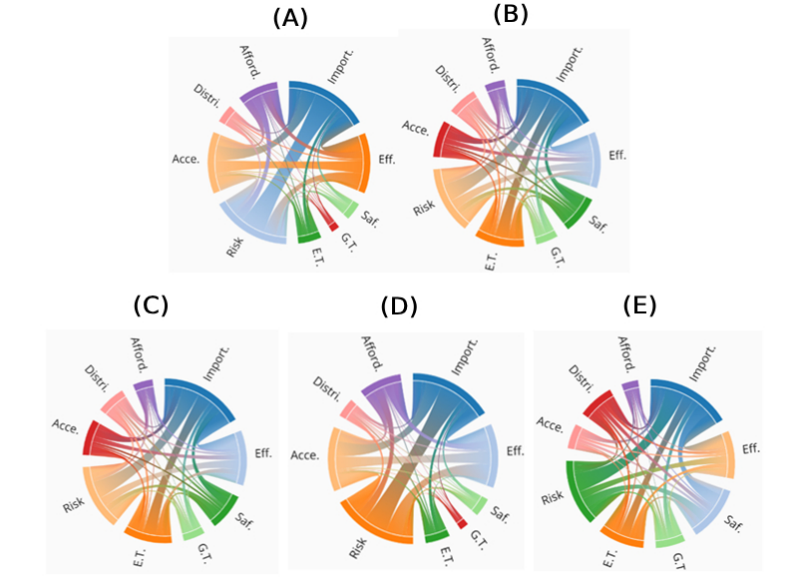
Comparison of agenda attribute interactions by different users: (A) government, (B) professional media, (C) alternative media, (D) civil societal organizations, (E) regular users. Acce.: vaccine accessibility, Afford.: vaccine affordability, Distri.: vaccine distribution, Eff.: vaccine effectiveness, E.T.: expert trust, G.T.: government trust, Import.: vaccine importance, Risk: risk of COVID-19, Saf.: vaccine safety.

To answer RQ2, this study computed the interrelationships between agenda attributes by the government and nongovernment users by constructing co-occurrence matrices. Results showed that “vaccine importance,” “vaccine effectiveness,” and “COVID-19 risk” were the most prominent attributes interacting with each other in the agendas of government and nongovernment users, except for the regular users’ agenda in which “vaccine safety” (n=2503) rather than “vaccine effectiveness” (n=2161) had more established connections overall with other attributes. Specifically, the government agenda featured strong connections between “vaccine importance” and “COVID-19 risk” (n=1505), followed by “vaccine importance” and “vaccine effectiveness” (n=945), “vaccine importance” and “accessibility” (n=940), and “COVID-19 risk” and “accessibility” (n=816). As for the agenda of professional media, the strongest link was established between “vaccine importance” and “COVID-19 risk” (n=1528), followed by the link between “vaccine importance” and “vaccine effectiveness” (n=1327) and the link between “vaccine importance” and “trust in experts” (n=1220). In terms of regular users, their agenda highlighted the relationships between “vaccine importance” and “COVID-19 risk” (n=931), “vaccine importance” and “vaccine effectiveness” (n=655), “vaccine importance” and “vaccine safety” (n=644), “vaccine importance” and “trust in experts” (n=536), and “vaccine safety” and “COVID-19 risk” (n=469). Using chord diagrams, this study visualized the interrelationships of agenda attributes by different user categories. The arc in the outer ring represents the attributes of the vaccination agenda and is differentiated by color. The arc length indicates the total number of associations an attribute maintains with other attributes when communicated by users in a specific category. The band within the ring represents the connected relationship between 2 topics, with the thickness of the band indicating the magnitude of the connection. A set of chord diagrams revealing agenda attribute interactions in the agendas with comparison of different users is presented in Figure 2.

To assess the evolution of links between attributes over time, this study also divided the co-occurrence dynamics of intragroup agenda attributes into 3 distinct periods: 2020, 2021, and 2022. Our findings revealed that the connections between agenda attributes varied by both the time period and the categories of Facebook users. Notably, in the government agenda, the link between “vaccine effectiveness” and “vaccine affordability” exhibited an increase in strength during 2022 (795/4150, 19.16%), compared with 2020 (18/223, 8.25%) and 2021 (690/4744, 14.54%). Conversely, the connection between “vaccine importance” and “expert trust” within the agenda of regular users demonstrated a decline in frequency over the 3-year span (2020: 119/1165, 10.21%; 2021: 282/3417, 8.25%; 2022: 118/1779, 6.63%). More information about the co-occurrence dynamics of the intragroup agenda attributes over time can be found in Multimedia Appendix 5.

### Agenda Network Analysis

In answering RQ3, the results of the QAP tests demonstrated significantly positive and strong correlations between the agenda network of the government and those of professional media (*r*=0.745, *P*=.005) and civil organizations (*r*=0.632, *P*=.02). However, the correlations between the government’s agenda network and the network of alternative media (*r*=0.462, *P*=.08) and regular users (*r*=.451, *P*=.07) were not statistically significant.

The subsequent QAP linear regression analysis tested whether the agenda network of the Macao government can predict that of nongovernment users. For example, by using the government as a predictor and different types of nongovernment users as outcome variables, the results demonstrated that the government has an impact on the agenda network of professional media (*b*=0.703, *P*=.006) and civil organizations (*b*=0.051, *P*=.02). The adjusted *R*^2^ value for professional media indicated that government accounts for around 54% of the variance in the professional media’s agenda network, while government only accounts for 38% of the variance in the agenda network of civil organizations. The results of the QAP linear regression analysis with the government as a predictor are shown in Table 2.

In the QAP linear regression model predicting the agenda of regular users, the results revealed significant impacts of alternative media (*b*=2.46, *P*=.001), professional media (*b*=0.52, *P*=.001), and civil organizations (*b*=6.16, *P*=.001) on the agenda of regular users. The adjusted *R*^2^ value for professional media, civil organizations, and alternative media ranged from 0.81 to 0.86, suggesting that all 3 categories of users can explain 81%-86% of the variance in the regular users’ agenda network. The results of the QAP linear regression analysis with regular users as the outcome variable are shown in Table 3.

**Table 2:** Quadratic assignment procedure regression analysis with government as the predictor.

User category	Unstandardized coefficient	*P* value^a^	*R*^2^ value	Adjusted *R*^*2*^
Civil organizations	0.051	.02	0.399	0.382
Professional media	0.703	.006	0.556	0.543
Alternative media	0.095	.10	0.214	0.191
Regular users	0.246	.12	0.204	0.180

^a^Outcomes were considered statistically significant at *P*<.05.

**Table 3:** Quadratic assignment procedure regression analysis with regular users as the outcome variable.

User category	Unstandardized coefficient	*P* value^a^	*R*^2^ value	Adjusted *R*^*2*^
Government	0.246	.12	0.204	0.180
Alternative media	2.462	.001	0.868	0.864
Professional media	0.521	.001	0.811	0.805
Civil organizations	6.164	.001	0.832	0.827

^a^Outcomes were considered statistically significant at *P*<.05.

### Impacts of Government and Nongovernment Users on Each Other’s Vaccine Agenda

To answer RQ4, the Granger causality test was further performed to examine whether the 9 attributes in the government’s agenda statistically predicted the future intensity of topics discussed by different categories of users and vice versa. Specifically, the results showed that attributes such as “vaccine safety” (*F*_3,13_=3.817; *P*=*.*04) and “trust in experts” (*F*_3,13_=3.916; *P*=.03) in the government’s agenda significantly affected such attributes in the agenda of nongovernment users, while the attributes associated with “trust in government” (*F*_3,13_=4.590; *P*=.02) and “vaccine affordability” (*F*_3,13_=3.851; *P*=.04) in the agenda of nongovernment users affected these attributes in the agenda of the government at the significance level of *P*<.05.

By classifying nongovernment users into different user categories, the results suggested a unidirectional trend in the attribute of “vaccine safety” flowing from the government’s agenda to that of professional media (*F*_5,15_=3.247; *P*=.03), while professional media affected the agenda of the government unilaterally through the attributes of “vaccine importance” (*F*_5,12_=7.192; *P*=.003), “vaccine effectiveness” (*F*_3,13_=4.391; *P*=.02), “COVID-19 risk” (*F*_5,15_=5.173; *P*=.006), and “vaccine affordability” (*F*_3,13_=4.754; *P*=.02). Additionally, alternative media affected the government by setting the agenda with attributes such as “COVID-19 risk” (*F*_5,15_=8.769; *P*<.001) and “vaccine accessibility” (*F*_5,15_=2.963; *P*=.047), while there was no temporal causation from the government to alternative media for the attributes identified.

Regarding civil organizations, the government predicted the agenda of civil organizations through the attributes of “vaccine importance” (*F*_5,15_=4.111; *P*=.01), “vaccine effectiveness” (*F*_3,13_=6.264; *P*=.007), and “trust in experts” (*F*_3,9_=15.877; *P*=.001), while the causation from civil organizations to the government was absent for all attributes except “vaccine safety” (*F*_3,12_=4.405; *P*=.03).

Most notably, the Granger causality analysis revealed that the government had a significant impact on the agenda of regular users through the attributes of “vaccine importance” (*F*_5,15_=3.809; *P*=.02), “trust in experts” (*F*_5,15_=16.639; *P*<.001), “vaccine accessibility” (*F*_5,15_=3.343; *P*=.03), and “vaccine affordability” (*F*_3,13_=6.012; *P*=.008). Despite the absence of Granger causality from regular users to the government for most attributes, there was a reciprocal relationship between the government and regular users in the attribute of “vaccine affordability.” The results of the Granger causality tests between the government and other types of users are shown in Table 4.

**Table 4:** Granger causality tests between government users and other types of users for each vaccine attribute.

Vaccine attribute		Nongovernment users			Professional media			Alternative media			Civil societal organizations			Regular users	
		Outcome variable	Antecedent variable	Outcome variable		Antecedent variable	Outcome variable		Antecedent variable	Outcome variable		Antecedent variable	Outcome variable		Antecedent variable
**Importance**															
	*F* value (df)	1.410 (5,20)	1.209 (2,20)	1.413 (5,20)		7.192 (5,12)	2.412 (5,15)		2.407 (5,15)	4.111 (5,15)		1.801 (5,15)	3.809 (5,15)		2.259 (5,15)
	*P* value	.26	.32	.26		.003	.09		.09	.01		.17	.02		.10
**Effectiveness**															
	*F* value (df)	0.449 (2,30)	3.029 (3,13)	0.133 (2,30)		4.391 (3,13)	0.293 (3,13)		1.319 (5,9)	6.264 (3,13)		0.567 (2,10)	0.968 (2,30)		0.858 (2,30)
	*P* value	.64	.07	.88		.02	.83		.34	.007		.58	.39		.44
**Safety**															
	*F* value (df)	3.817 (3,13)	3.222 (3,13)	3.247 (5,15)		2.565 (5,15)	0.706 (1,15)		2.419 (5,15)	2.923 (3,13)		4.405 (3,12)	2.004 (5,15)		2.912 (3,22)
	*P* value	.04	.057	.03		.07	.41		.08	.07		.03	.14		.057
**Trust in government**															
	*F* value (df)	2.017 (3,15)	4.590 (3,13)	3.270 (3,13)		3.924 (3,13)	1.228 (2,20)		2.296 (3,13)	2.705 (3,9)		3.585 (2,10)	0.304 (2,10)		3.373 (3,13)
	*P* value	.15	.02	.055		.03	.31		.12	.11		.07	.74		.051
**Trust in experts**															
	*F* value (df)	3.916 (3,13)	0.402 (2,20)	3.753 (2,10)		1.437 (5,30)	0.401 (2,20)		1.146 (2,20)	15.877 (3,9)		1.058 (1,22)	16.639 (5,15)		4.189 (2,9)
	*P* value	.03	.67	.06		.24	.67		.34	.001		.31	<.001		.051
**COVID-19 risk**															
	*F* value (df)	0.255 (2,9)	1.124 (2,30)	1.890 (2,30)		5.173 (5,15)	0.665 (3,3)		8.769 (5,15)	2.442 (3,20)		2.275 (3,20)	0.655 (2,15)		0.235 (2,15)
	*P* value	.78	.34	.16		.006	.63		<.001	.09		.11	.53		.79
**Accessibility**															
	*F* value (df)	0.248 (2,15)	2.781 (3,13)	0.045 (2,10)		1.362 (5,20)	1.461 (5,15)		2.963 (5,15)	1.546 (5,15)		2.763 (5,10)	3.343 (5,15)		2.376 (5,15)
	*P* value	.78	.08	.96		.28	.26		.047	.23		.08	.03		.09
**Distribution**															
	*F* value (df)	0.756 (2,20)	0.104 (2,20)	0.596 (2,20)		0.283 (2,20)	0.147 (1,25)		0.005 (1,15)	1.264 (1,20)		0.382 (1,20)	4.175 (1,25)		0.458 (1,25)
	*P* value	.48	.90	.56		.76	.70		.94	.27		.54	.051		.50
**Affordability**															
	*F* value (df)	2.500 (3,13)	3.851 (3,13)	0.745 (2,20)		4.754 (3,13)	0.479 (2,20)		0.688 (2,20)	0.525 (2,20)		0.495 (2,20)	6.012 (3,13)		5.067 (2,20)
	*P* value	.10	.04	.49		.02	.63		.51	.60		.62	.008		.02

## Discussion

### Principal Findings

This study examined the dynamics and patterns of vaccine communication on Facebook in Macao during the COVID-19 pandemic. The principal findings demonstrated that “vaccine importance” was the most prevalent attribute in the vaccination agenda on Facebook, followed by the attributes of “COVID-19 risk” and “trust in experts.” The overall vaccination agenda revealed the highest co-occurrences were between “vaccine importance” and “COVID-19 risk.” Differences existed in agenda priorities between the government and regular users. The government primarily focused on the risks of COVID-19 and the effectiveness of vaccines, whereas regular users were more concerned with the safety and distribution of vaccines. The Macao government played a role in shaping the agenda for regular users by highlighting vaccine importance (Granger causality result: *F*_5,15_=3.809; *P*=.02), trust in experts (Granger causality result: *F*_5,15_=16.639; *P*<.001), and vaccine accessibility (Granger causality result: *F*_5,15_=3.343; *P*=.03) and affordability (Granger causality result: *F*_3,13_=6.012; *P*=.008), while its impact on the agenda network of regular users remained insignificant (QAP result: *b*=0.246; *P*=.12). Both government and nongovernment users (eg, professional media, alternative media, civil organizations, and regular users) had intertwined agendas with mutual influence.

Unlike previous studies that predominantly focused on single aspects of vaccine communication (eg, [17,34]), this study used a more holistic approach to reveal the role of various actors including the government, professional media, alternative media, civil organizations, and regular users in promoting vaccination agendas and the interplay of diverse actors in the vaccine agenda setting process. The results of this study suggest that professional media acts as more than simple information providers to the government but rather effectively pushed agenda setting as a supplementary process to vaccine promotion by raising salient topics that the government fails to identify due to lack of information and experience. The government, however, is more likely to respond to professional media to receive timely feedback on vaccination issues for the purpose of learning and improvement. This can be observed from the impact that professional media has on the government in the agenda setting process through topics of “vaccine importance” (Granger causality results: *F*_5,12_=7.192; *P*=.003), “vaccine effectiveness” (Granger causality results: *F*_3,13_=4.391; *P*=.02), “trust in government” (Granger causality results: *F*_3,13_=3.924; *P*=.03), “COVID-19 risk” (Granger causality results: *F*_5,15_=5.173; *P*=.006), and vaccine affordability (Granger causality results: *F*_3,13_=4.754; *P*=.02).

### Who Leads the Vaccine Agenda of Whom?

Despite a significant correlation between the government agenda network and the agenda network of nongovernment users, the government had a limited impact on the agenda attributes of different Facebook user categories and vice versa. As Facebook is an open platform where information from a wide variety of sources freely circulates and interacts, it is difficult to determine the driving force behind the vaccine promotion agenda on the platform [55]. In other words, nongovernment users’ vaccine promotion agendas may have been impacted by other sources, such as the World Health Organization or other health professionals, which indicates a multidirectional effect.

As such, it appears that the government did not unilaterally set the agenda of nongovernment users. Instead, there is a “2-way” interaction between government and nongovernment user agendas. Due to their mutual effect, neither the government nor nongovernment users lead the agenda on social media. It is likely that the government and different types of nongovernment users pay attention to the agendas of one another and interact with one another to build the overall vaccine agenda network on Facebook. This corresponds with the argument by Finset et al [63] that, amid the near-chaotic flow of information, every individual, in different roles and with varied responsibilities, can contribute to the development of the information flow and agenda on COVID-19. A plausible explanation for this outcome could be the unprecedented nature of the health crisis. The lack of up-to-date crisis communication planning and experience with coping with a novel crisis may challenge the government’s agenda-setting process, particularly in terms of vaccine promotion.

### Comparison With Prior Work

Previous agenda setting research found that changes in the government agenda led to changes in the public agenda [64]. However, during the COVID-19 pandemic, the public was no longer passive consumers of social media. Our results indicating the different concerns of vaccination between the government and regular users corroborate previous findings by Zhou and Zheng [44] who found that, during the COVID-19 pandemic, the government’s Weibo account exhibited a more propaganda-oriented approach, whereas public accounts were more attentive to issues that directly pertained to self-interest, such as protective measures against the virus and minimizing financial losses. Unlike other political issues, the government may have less impact on shaping public agenda due to the more collected information possessed by the public. This is partly consistent with some recent research indicating that shaping public opinion in a fragmented digital environment such as social media is challenging [54,65]. Additionally, the case of Macao also indicates selective public responsiveness on topics that are clear and straightforward, which partially verifies the observation by Kim [66] that individuals are more receptive to topics that are unambiguous and do not demand extensive background knowledge as they may not have enough background information with which to fully process any new information on complex topics.

### Practical Implications

Our study provides several implications to inform the management of future pandemics. First, given the disparity between the government and public agenda networks, it is crucial to bridge the gap to enable effective vaccine communication. Policymakers should strive for alignment between government messaging and public concerns, addressing issues that are prominent within the public discourse. Social media listening activities are invaluable tools for understanding public health concerns. By monitoring public conversation through social media listening, policymakers can develop targeted messaging and communication strategies that effectively address public concerns and provide accurate information to dispel misconceptions.

Second, the low responsiveness of the public agenda to the government agenda indicates the need to enhance the government impact on the public agenda. Governments can streamline their messaging by using plain language, which helps individuals with different levels of knowledge understand information easily. Clear and concise presentation avoids unnecessary complexity. Visual aids and interactive media can also be used to improve public involvement and responsiveness, overcoming barriers caused by limited background information.

Third, policymakers’ efforts to convince the public to receive vaccines in response to potential health risks have been shown in our study to lead to a spillover of media attention that significantly drives the vaccination agenda among the public. Collaboration with influential media, including professional and alternative media, thus offers a powerful means to facilitate vaccination policy and improve public health. Governments can utilize the extensive reach and persuasive power of media outlets to actively involve and inform the public about specific issues that should receive priority attention, thereby advancing the government’s crisis management initiatives.

Fourth, civil organizations’ ability to shape public attention toward vaccination issues by influencing the public agenda network suggests that their impact on shaping the vaccination agenda may be underestimated or overlooked. Driven by social responsibility, civil organizations often dedicate their efforts to promoting public health by increasing awareness and advocating for public health policies [43]. The close ties to communities enable them to be trusted sources of information for the public. Therefore, through partnerships with civil organizations, governments can leverage their networks, expertise, and community trust to effectively promote vaccination initiatives.

### Limitations

Several limitations warrant consideration. First, broadening the scope beyond vaccines to encompass diverse policies could offer a more comprehensive understanding of public attention allocation mechanisms. Researchers are encouraged to explore various policies to enhance generalizability. Second, although Facebook data provided valuable insights, the findings are platform-specific and may not apply universally. Future studies should incorporate a diverse set of social media platforms and combine quantitative data with surveys and interviews for a more nuanced perspective. Third, although this study explored temporal agenda dynamics, it did not delve into the determinants driving public attention intensity, such as government transparency and issue salience. Investigating these factors could provide valuable insights into the agenda setting process at the government level.

### Conclusions

This study investigated the communication dynamics of COVID-19 vaccines in Macao, with a specific focus on how government agendas impact other entities on Facebook. Our results reveal that the Macao Government’s efforts to set the vaccination agenda on Facebook have shown limited effectiveness in shaping the public’s discourse and priorities regarding vaccines. Such findings have profound implications for shaping government responses to future pandemics. Authorities, in their endeavor to legitimize policies, must recognize the intricate interplay between their agendas and public reception. Although agenda setting serves as a strategic tool to promote vaccination, it also exhibits limitations. This requires a shift toward more nuanced, strategy-focused research. This study offers indispensable insights in the area of crisis communication, underscoring the urgent necessity of bridging the gap between government and public agendas. Furthermore, it illuminates the potential of collaborations with influential media outlets and civil organizations as formidable channels to augment the reach and influence of vaccination agendas set by the government.

## Appendix

Multimedia Appendix 1 Keywords for vaccine-related data acquisition.

Multimedia Appendix 2 Coding framework for COVID-19 vaccine posts on Facebook.

Multimedia Appendix 3 Keywords for machine coding of vaccine-related topics.

Multimedia Appendix 4 Outcomes of post hoc tests on the significant difference between user categories and vaccine-related topics.

Multimedia Appendix 5 Intra-group co-occurrences dynamics of agenda attributes for the years of 2020, 2021, and 2022.

## Abbreviations

NAS: network agenda setting model
QAP: quadratic assignment procedure
RQ: research question
VAR: vector autoregression
