# Supplementary material for: Government-Nongovernmental Organization (NGO) Collaboration in Macao’s COVID-19 Vaccine Promotion: Social Media Case Study
Source: JMIR Infodemiology. 2024 Mar 19;4:e51113. doi: 10.2196/51113 (PMC10988378; doi:10.2196/51113)
Supplement: Multimedia Appendix 1 [file infodemiology_v4i1e51113_app1.docx]

# Appendix 1. Keywords for vaccine-related data acquisition

| Topics | Keyword Combination |
| --- | --- |
| COVID-19-related terms | "武漢不明原因肺炎" OR "新冠肺炎疫情" OR "武漢肺炎" OR "新型冠狀病毒" OR "疫情" OR "新冠疫情" OR "抗疫" OR "防疫" OR "新冠病毒" OR "武漢病毒" OR "中國病毒" OR "功夫流感" OR "COVID-19" OR "novel coronavirus" OR "2019-ncov" OR "NCP" OR "Novel Corona Pneumonia" OR "Chinese virus" OR "SARS-CoV-2" OR "pandemic" OR "epidemic" OR "武漢病毒" OR "Wuhan virus" OR "Wuhan pneumonia" OR "中國肺炎" OR "China pneumonia" OR "中國人肺炎" OR "Chinese pneumonia" |
| Vaccine-related terms | “疫苗” OR “打針” OR “谷針” OR “逼針” OR “國藥” OR “科興” OR “復星- BioNTech” OR “滅活疫苗” OR “m-RNA 疫苗” |
