# Supplementary material for: Government-Nongovernmental Organization (NGO) Collaboration in Macao’s COVID-19 Vaccine Promotion: Social Media Case Study
Source: JMIR Infodemiology. 2024 Mar 19;4:e51113. doi: 10.2196/51113 (PMC10988378; doi:10.2196/51113)
Supplement: Multimedia Appendix 2 [file infodemiology_v4i1e51113_app2.docx]

# Appendix 2. Coding framework for COVID-19 vaccine posts with examples

| Topics | Definition | Example |
| --- | --- | --- |
| Vaccine  Importance | Indicate that COVID-19 vaccine is important, necessary or needed. | “「新冠疫情」自爆發以來，改變了各國人民的正常生活和往來，疫苗則可以挽救無數人的生命，並抑制「疫情」的傳播。澳門能在如此短的時間內獲得疫苗，並開始為高危人群接種，從科學角度來說著實意義非凡。”  “Since the outbreak of COVID-19, the normal life of people around the world have been changed. Vaccines can save countless lives and curb the spread of COVID-19. From a scientific point of view, it is remarkable that Macao has been able to obtain the vaccine in such a short period of time and to start vaccinating high-risk groups.”  ——By陳家良on February 3, 2021 |
| Vaccine  Effectiveness | Indicate that COVID-19 vaccine is effective, able to produce antibodies or to prevent COVID-19, etc. | “……上述結果顯示，成年人接種經批准的mRNA疫苗，能夠有效預防感染，且可降低突破性感染個案的病毒載量、發燒風險及縮短病程，但也進一步證明衛生當局持續強調的說法，即接種疫苗不能完全預防感染，不過症狀相對會較為溫和。”  “The results suggest that adults receiving an approved mRNA vaccine can effectively prevent infection and reduce fever risk and disease duration in breakthrough infections, but add to what health authorities continue to emphasize that vaccination does not completely prevent infection, but that symptoms are relatively mild.”  ——By Macao Daily News on July 1, 2021 |
| Vaccine  Safety | Indicate that COVID-19 vaccine is safe, or without severe side-effects or adverse reactions, etc. | “目前，國藥集團中國生物新冠疫苗3至17歲年齡組已經在河南完成Ⅰ/Ⅱ期臨床試驗……接種後安全性良好，不良反應主要為發熱和接種部位疼痛，嚴重程度以1級為主，未見嚴重不良反應；不良反應發生率隨接種劑次增加而逐漸降低。”  “At present, the Sinopharm Group has completed phase Ⅰ/Ⅱ clinical trials of its Biological novel coronavirus vaccine in the 3-17 year old group in Henan Province…The adverse reactions were mainly fever and pain at the site of inoculation. The severity was mainly grade 1, and no serious adverse reactions were observed. The incidence of adverse reactions decreased with increasing dose”  —— By Macao Daily News on July 16, 2021 |
| Trust in  Governments | Indicate trust in government or policy-makers including all-level government, ministry of health, CDC, etc. | “市民普遍信任政府在衛生防護的專業能力，積極配合政府接種疫苗防疫。”  “Citizens generally trust the government's professional competence in health protection and cooperate with the government to vaccinate for disease prevention.”  ——By訊報 on July 28, 2021 |
| Trust in  Experts | Indicate trust in experts or professional organization in the field of epidemic prevention (e.g., universities, research institutes, etc). | “根據世衛的資料顯示，已接種2劑滅活疫苗的人士，若第3劑選擇mRNA疫苗的話，其效果會較好；國家疾病預防控制中心早前也指出，倘已接種2劑滅活疫苗的人士，第3劑轉而接種其他技術路線的疫苗，所產生的免疫效果會較好。此外，香港的衛生部門也公佈，已接種2劑疫苗的人士，無論是滅活疫苗或mRNA疫苗，香港的專家建議第3劑均改為接種mRNA疫苗。因此，已接種2劑疫苗的市民，可考慮接種mRNA疫苗作為第3劑加強劑。”  “According to WHO, people who have received two doses of inactivated vaccine will be more effective if they choose mRNA vaccine for the third dose. Chinese CDC also noted earlier that for people who have received two doses of the inactivated vaccines, the immune effect will be better if they receive the third dose of other types of vaccines. In addition, Hong Kong's Health Department also announced that people who have received two doses of vaccine, whether inactivated vaccine or mRNA vaccine, are suggested to receive the third dose of mRNA vaccine. Therefore, people who have received two doses of COVID-19 vaccines can consider to receive the mRNA vaccine as the third booster dose.  ”  By梳打埠 on January 7, 2022 |
| High Risk of COVID-19 | Indicate that COVID-19 is susceptible and severe if suffered, or COVID-19 epidemic is severe and fearful. | “市民應留意疫情發展，嚴格執行各項防疫措施，保持距離，避免人流聚集, 產生感染風險…疫苗可有效預防新冠病毒肺炎，有效減少自身感染、重症和死亡風險，築起免疫屏障，保護自己及家人。”  “Citizens should pay close attention to the development of the epidemic, strictly implement various prevention measures, maintain social distance to avoid the risk of infection... Vaccines can effectively prevent COVID-19, reduce the risk of infection, severe illness and death, and build an immune barrier to protect yourself and your family.”  —— By Macao Daily News on November 5, 2021 |
| Vaccine Accessibility | Mention production or supply capacity of COVID-19 vaccine. | “新型冠狀病毒感染應變協調中心表示，至目前爲止，共購入了130萬劑的國藥疫苗和40萬劑mRNA疫苗，已經使用了92萬劑國藥疫苗和20萬劑mRNA疫苗。當局正準備再購入38萬劑國藥疫苗和20萬劑mRNA疫苗。以上疫苗的數量足夠供應澳門市民接種。”  “According to the COVID-19 response Coordination Center, 1.3 million doses of Sinopharm-produced vaccines and 400,000 doses of mRNA vaccines have been purchased so far... The authority is preparing to buy another 380,000 doses of Sinopharm-produced vaccines and 200,000 doses of mRNA vaccines. The above quantity of vaccine is sufficient to supply to Macao citizens.”  By News Bureau of the Macao Government on March 3, 2022 |
| Vaccine Distribution | Mention priority vaccination groups, compulsory or voluntary vaccination. | “部份較早前已接種第3劑新冠疫苗的人士，至今距離接種期已有一段時間，按照不同地方經驗，稍後將考慮為風險特別高的人士，例如長者，推出優先接種第4劑新冠疫苗計劃。”  “For those who received the third dose of COVID-19 vaccine earlier, it has been some time since the vaccination period began. According to previous experience, priority for the fourth dose of COVID-19 vaccine will be considered later for those at particularly high risk, such as the elderly.”  By颱風及民防訊息發佈 (Typhoon and civil defence information released) on July 4, 2022 |
| Vaccine Affordability | Mention prices of COVID-19 vaccine, free or not. | “澳門政府免費為外勞接種！！！外勞一元都唔洗俾”  “The Macao government provides free vaccination service to foreign workers!! They donot need to pay even one dollar!”  By Sam Car on January 25, 2021 |
| Facebook Users Classification | | |
| Government Accounts | All authorities which serve as the channel of official information release during the COVID-19. | News Bureau of the Macao government (澳門特區政府新聞局);  Special Webpage against Epidemic - Centre for Disease Control and Prevention (新型冠狀病毒感染應變協調中心信息發佈專頁) |
| Civil Society  Organization Accounts | Organizations or associations which are established by individuals or groups with a common purpose or interest and operate in the community, differing from the government and corporations. | Macau Youth Volunteers Association (澳門青年志願者協會) |
| Professional Media  Accounts | Also known as mainstream media, including print media and broadcast media which are responsible for information dissemination and public awareness. | Macao Daily (澳門日報);  TDM - Teledifusao De Macao (澳廣視新聞) |
| Alternative media Accounts | Media sources that are independent and distinct from [mainstream mass media](https://en.wikipedia.org/wiki/Mainstream_media) in terms of their content, production, or distribution. Alternative media characterizes in more freedom of press. | Macao Concealers (愛瞞日報); All about Macao |
| Regular user Accounts | Individual Facebook users | - |

*Note*. this coding framework is partly adapted from Hou and colleagues (2021).
