# Supplementary material for: Government-Nongovernmental Organization (NGO) Collaboration in Macao’s COVID-19 Vaccine Promotion: Social Media Case Study
Source: JMIR Infodemiology. 2024 Mar 19;4:e51113. doi: 10.2196/51113 (PMC10988378; doi:10.2196/51113)
Supplement: Multimedia Appendix 3 [file infodemiology_v4i1e51113_app3.docx]

# Multimedia Appendix 3. Keywords for machine coding of vaccine-related topics

| **Vaccine Topics** | | | **Keywords** |
| --- | --- | --- | --- |
| Vaccine Importance |  | 疫苗 AND ((預防 OR 有利於 OR 最大限度 OR 發揮作用 OR 減少 OR 消除 OR 抗體 OR 保護 OR 重要 OR 重中之重 OR 關鍵 OR 至關重要 OR 必不可少 OR 不可缺少 OR 不可替代 OR 無可替代) OR ((降低 OR 減少) AND (風險 OR 概率)))  Vaccines AND ((Prevent OR benefit OR maximize OR function OR reduce OR eliminate OR antibody OR protection OR important OR priority OR critical OR essential OR indispensable OR irreplaceable OR irreplaceable) OR ((reduce OR reduce) AND (risk OR probability)) | |
| Vaccine Effectiveness |  | 疫苗 AND (有效 OR 有用 OR 靈驗 OR 靈 OR 高效)  Vaccines AND (effective OR useful OR efficacious OR effective) | |
| Vaccine Safety |  | 疫苗 AND ((安全 OR 可靠) OR (少 AND (風險 OR 副作用 OR 中風 OR 歪嘴 OR 不良反應 OR 面癱)))  Vaccine AND ((safe OR reliable) OR (less risk OR side effects OR stroke OR crooked mouth OR adverse reactions OR facial paralysis)) | |
| Government Trust |  | 疫苗 AND ((相信 OR 信賴 OR 堅信 OR 肯定 OR 確信 OR 信任) AND (中央 OR 政府 OR 特首 OR 賀一誠 OR 衛生局 OR 李展潤 OR 羅奕龍 OR 習近平))  Vaccine AND ((believe OR trust OR believe OR affirm OR believe OR trust) AND (Central Government OR Chief Executive OR Ho Iat Seng OR Health Bureau OR Xi Jinping)) | |
| Expert Trust |  | (專家 OR 教授 OR 大學 OR 研究所 OR 研究 OR 實驗 OR 研究機構 OR 實驗室 OR 醫生 OR 學者 OR 權威 OR 研究團隊 OR 科學家 OR 博士 OR 研究人員) NOT (假 OR 陰謀 OR 隱瞞 OR 欺騙 OR 厄 OR 呃 OR 誤導 OR 不可信 OR 不值得相信 OR 不可靠 OR 不靠譜 OR 唔靠譜 OR 靠唔住 OR 唔靠得住 OR 無說服力 OR 不要相信 OR 別信 OR 唔好信 OR 唔好相信 OR 咪信)  (Expert OR professor OR university OR institute OR research OR experiment OR research institution OR laboratory OR doctor OR scholar OR authority OR research team OR scientist OR doctor OR researcher) NOT (false OR conspiracy OR concealment OR concealment or concealment Deceive OR err OR mislead OR be unreliable OR untrustworthy OR unconvincing OR do not believe) | |
| Risk of COVID-19 |  | (新冠肺炎 OR 新冠病毒 OR 病毒 OR "COVID-19" OR 武漢肺炎 OR 新冠 OR 新型冠狀病毒 OR 疫情) AND ((重症 OR 死 OR 重病 OR 嚴重 OR 危 OR 不治 OR 創傷 OR 後遺症) OR (易 感染~5) OR (傳播速度 快~5) OR (易 中招~5) OR (易 傳染~5) OR (易 傳播~5) OR 高風險 OR (風險 高~5) OR ("唔是普通流感" OR "唔是大號流感" OR "不是普通流感" OR "不是大號流感"))  (Novel coronavirus pneumonia OR novel coronavirus OR virus OR "COVID-19" OR Wuhan pneumonia OR epidemic) AND ((severe OR death OR serious OR serious OR critical OR no treatment OR trauma OR sequelae) OR (susceptible to infection ~5) OR (fast spreading ~5) OR (susceptible to infection ~5) OR (susceptible to infection ~5) OR (susceptible to transmission ~5) OR high risk OR (high risk ~5) OR (" not normal flu "OR" not large flu "OR" not normal flu "OR "Not the big flu ")) | |
| Vaccine accessibility |  | 疫苗 AND (充足 OR 充裕 OR 可以保證 OR 足夠 OR 可以滿足 OR 能夠滿足)  Vaccine AND (sufficient OR sufficient OR can be guaranteed OR sufficient OR can be satisfied OR can be satisfied) | |
| Vaccine distribution |  | 疫苗 AND (優先 OR 強制 OR 自願)  Vaccine AND (priority OR mandatory OR voluntary) | |
| Vaccine affordability |  | 疫苗 AND (免費 OR 唔使錢 OR 政府出錢)  Vaccines AND (free OR at no cost) | |

# *Note.* The English translation for keywords employed are provided for reference.
