# Supplementary material for: Government-Nongovernmental Organization (NGO) Collaboration in Macao’s COVID-19 Vaccine Promotion: Social Media Case Study
Source: JMIR Infodemiology. 2024 Mar 19;4:e51113. doi: 10.2196/51113 (PMC10988378; doi:10.2196/51113)
Supplement: Multimedia Appendix 4 [file infodemiology_v4i1e51113_app4.docx]

# Appendix 4. Outcomes of post hoc tests on the significant difference between user categories and vaccine-related topics

| Topic | |  | Government | | Professional Media | Alternative Media | Civil Organization | Regular User |
| --- | --- | --- | --- | --- | --- | --- | --- | --- |
| Vaccine Importance | Count | | | 1616 | 2931 | 697 | 298 | 1816 |
|  | Expected Count | | | 1582 | 2931.4 | 754.8 | 270.6 | 1819.2 |
|  | % within User | | | 30.36 | 38.8 | 24.77 | 32.46 | 28.48 |
|  | Adjusted Residual | | | 1.087 | -0.009 | -2.503 | 1.911 | -0.098 |
|  | *P* value^a^ | | | 0.27704 | 0.99282 | 0.01231 | 0.056 | 0.92193 |
| Vaccine Effectiveness | Count | | | 1003 | 1638 | 404 | 181 | 937 |
|  | Expected Count | | | 895.1 | 1658.5 | 427 | 153.1 | 1029.3 |
|  | % within User | | | 18.85 | 21.68 | 14.36 | 19.72 | 14.69 |
|  | Adjusted Residual | | | 4.342 | -0.692 | -1.255 | 2.448 | -3.535 |
|  | *P* value^a^ | | | 0.00001 | 0.48894 | 0.20948 | 0.01437 | 0.00041 |
| COVID-19 Risk | Count | | | 1805 | 2651 | 681 | 211 | 1529 |
|  | Expected Count | | | 1478.6 | 2739.7 | 705.4 | 252.9 | 1700.3 |
|  | % within User | | | 33.92 | 35.09 | 24.2 | 22.98 | 23.98 |
|  | Adjusted Residual | | | 10.706 | -2.442 | -1.086 | -3.003 | -5.35 |
|  | *P* value^a^ | | | 0.00000 | 0.01461 | 0.27748 | 0.00267 | 0.00000 |
| Vaccine Safety | Count | | | 393 | 1374 | 359 | 140 | 1092 |
|  | Expected Count | | | 722 | 1337.8 | 344.5 | 123.5 | 830.2 |
|  | % within User | | | 7.38 | 18.19 | 12.76 | 15.25 | 17.12 |
|  | Adjusted Residual | | | -14.545 | 1.343 | 0.87 | 1.591 | 11.02 |
|  | *P* value^a^ | | | 0.00000 | 0.17927 | 0.3843 | 0.11161 | 0.00000 |
| Government Trust | Count | | | 133 | 752 | 175 | 40 | 493 |
|  | Expected Count | | | 342.5 | 634.6 | 163.4 | 58.6 | 393.9 |
|  | % within User | | | 2.5 | 9.95 | 6.22 | 4.36 | 7.73 |
|  | Adjusted Residual | | | -13.082 | 6.15 | 0.98 | -2.534 | 5.896 |
|  | *P* value^a^ | | | 0.00000 | 0.00000 | 0.32709 | 0.01128 | 0.00000 |
| Expert Trust | Count | | | 518 | 1895 | 593 | 154 | 1160 |
|  | Expected Count | | | 928.8 | 1721 | 443.1 | 158.9 | 1068.1 |
|  | % within User | | | 9.73 | 25.08 | 21.07 | 16.78 | 18.19 |
|  | Adjusted Residual | | | -16.266 | 5.78 | 8.034 | -0.423 | 3.466 |
|  | *P* value^a^ | | | 0.00000 | 0.00000 | 0.00000 | 0.6723 | 0.00053 |
| Vaccine Accessibility | Count | | | 1010 | 981 | 196 | 80 | 416 |
|  | Expected Count | | | 576.9 | 1068.9 | 275.2 | 98.7 | 663.3 |
|  | % within User | | | 18.98 | 12.98 | 6.97 | 8.71 | 6.52 |
|  | Adjusted Residual | | | 21.194 | -3.609 | -5.249 | -1.995 | -11.526 |
|  | *P* value^a^ | | | 0.00000 | 0.00031 | 0.00000 | 0.04604 | 0.00000 |
| Vaccine Affordability | Count | | | 605 | 529 | 128 | 53 | 370 |
|  | Expected Count | | | 362.3 | 671.3 | 172.8 | 62 | 416.6 |
|  | % within User | | | 11.37 | 7 | 4.55 | 5.77 | 5.8 |
|  | Adjusted Residual | | | 14.757 | -7.26 | -3.692 | -1.191 | -2.698 |
|  | *P* value^a^ | | | 0.00000 | 0.00000 | 0.00022 | 0.23365 | 0.00698 |
| Vaccine Distribution | Count | | | 341 | 1005 | 309 | 113 | 724 |
|  | Expected Count | | | 535.8 | 992.8 | 255.6 | 91.7 | 616.1 |
|  | % within User | | | 6.41 | 13.3 | 10.98 | 12.31 | 11.35 |
|  | Adjusted Residual | | | -9.861 | 0.519 | 3.658 | 2.358 | 5.2 |
|  | *P* value^a^ | | | 0.00000 | 0.60376 | 0.00025 | 0.01837 | 0.00000 |

*Note*. ^a^ Bonferroni *P* value was applied to correct for multiple comparisons. Outcomes are considered statistically significant at *P* < .001.
