# Supplementary material for: Government-Nongovernmental Organization (NGO) Collaboration in Macao’s COVID-19 Vaccine Promotion: Social Media Case Study
Source: JMIR Infodemiology. 2024 Mar 19;4:e51113. doi: 10.2196/51113 (PMC10988378; doi:10.2196/51113)
Supplement: Multimedia Appendix 5 [file infodemiology_v4i1e51113_app5.docx]

Appendix 5: Intra-group co-occurrences dynamics of agenda attributes for the years of 2020, 2021 and 2022

**Government**:

| 2020 | 2021 | 2022 |
| --- | --- | --- |
| 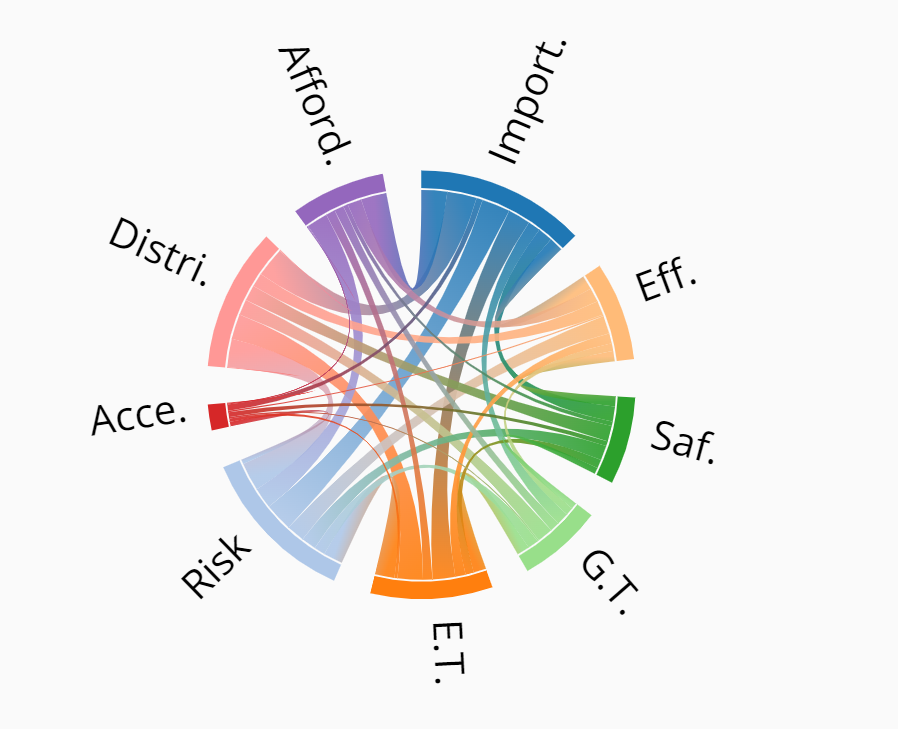 | 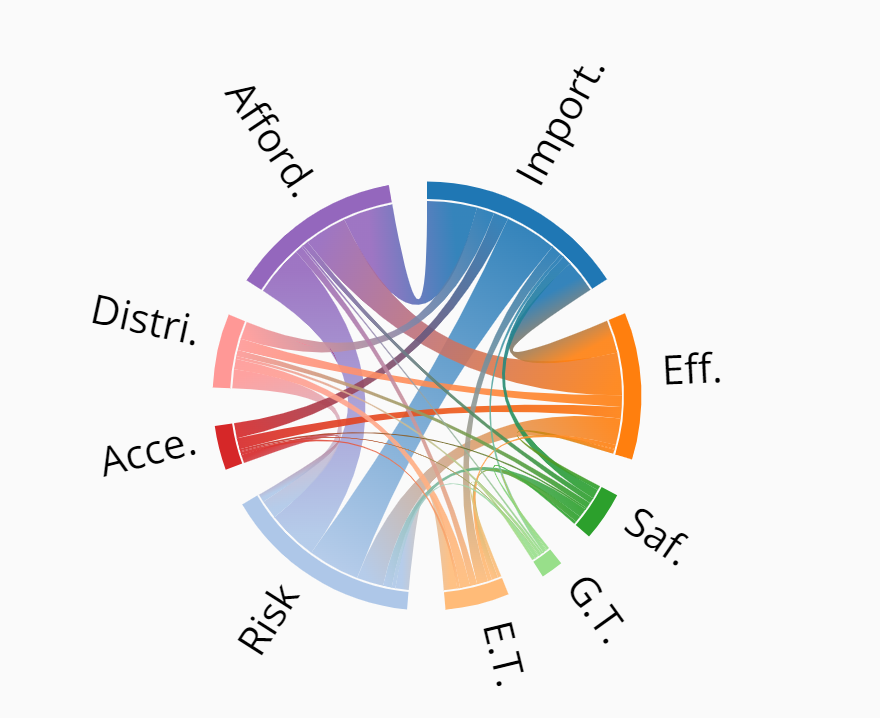 | 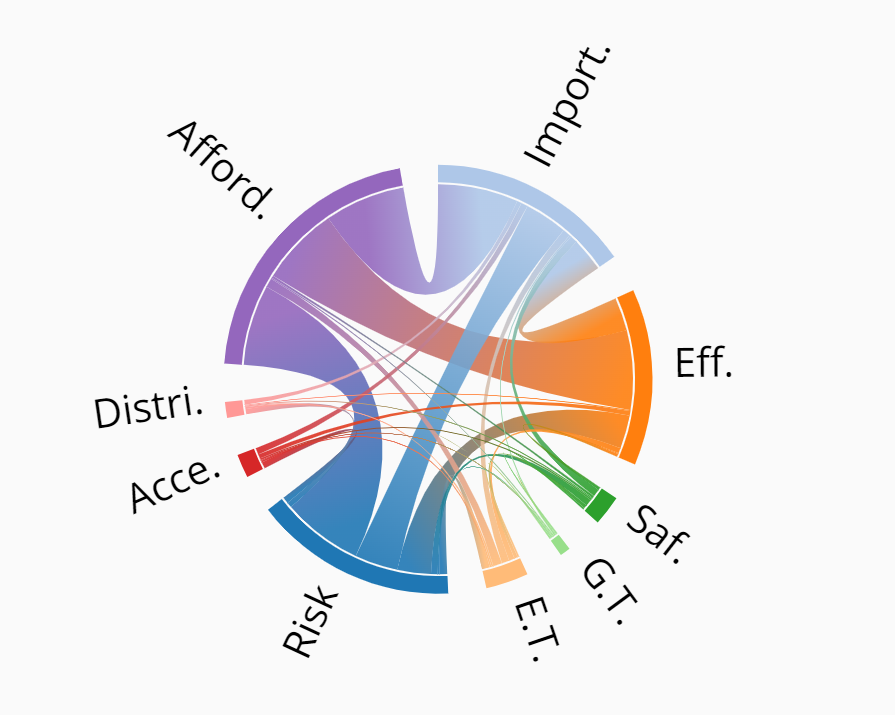 |

**Professional media:**

| 2020 | 2021 | 2022 |
| --- | --- | --- |
| 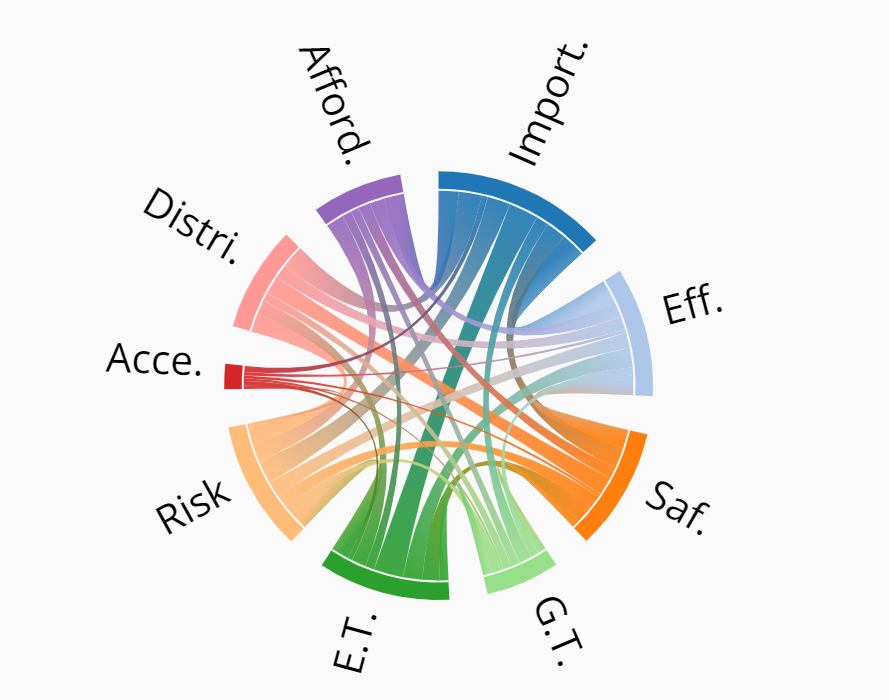 | 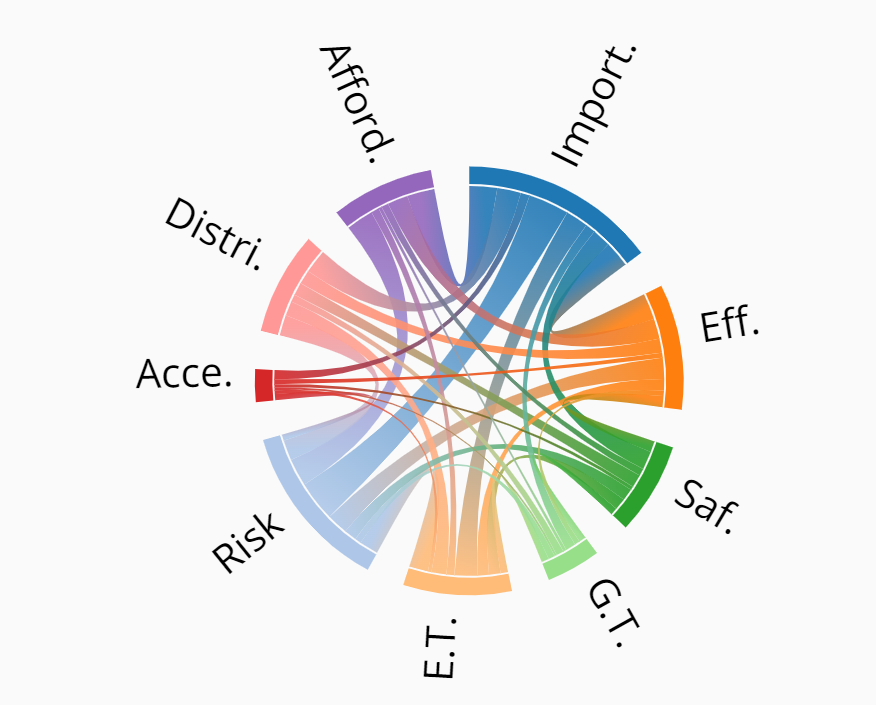 | 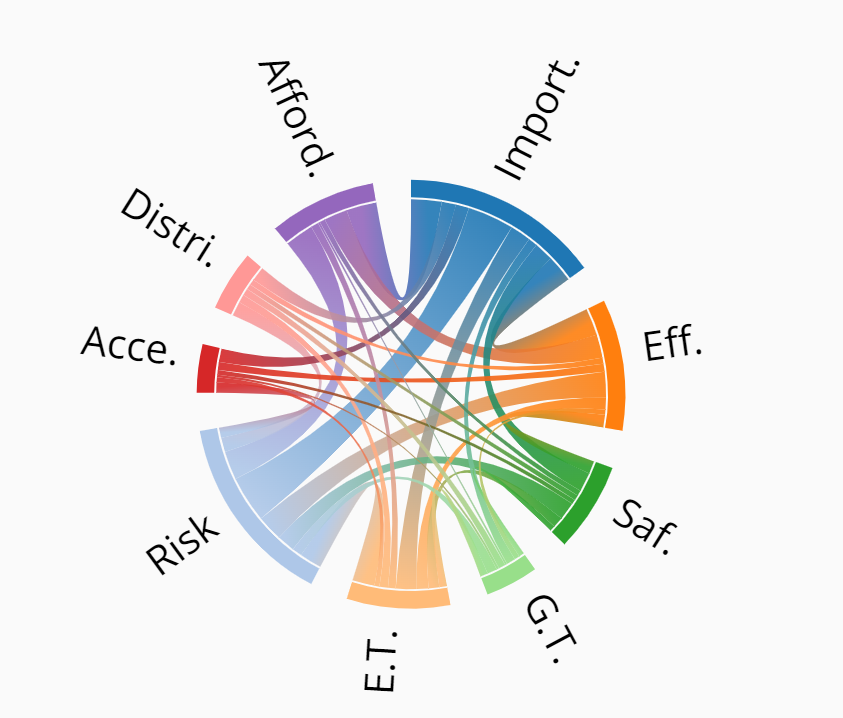 |

| 2020 | 2021 | 2022 |
| --- | --- | --- |
| 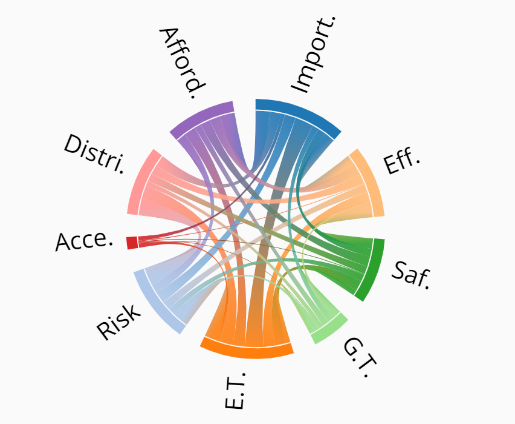 | 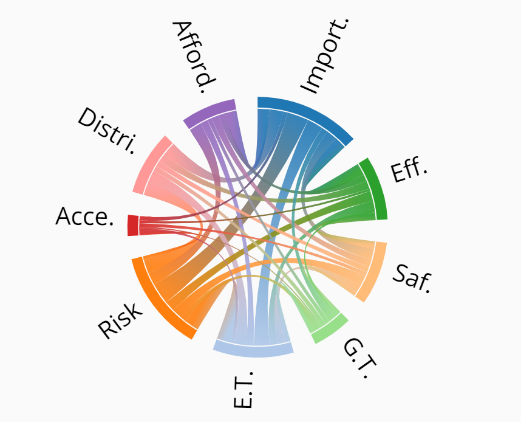 | 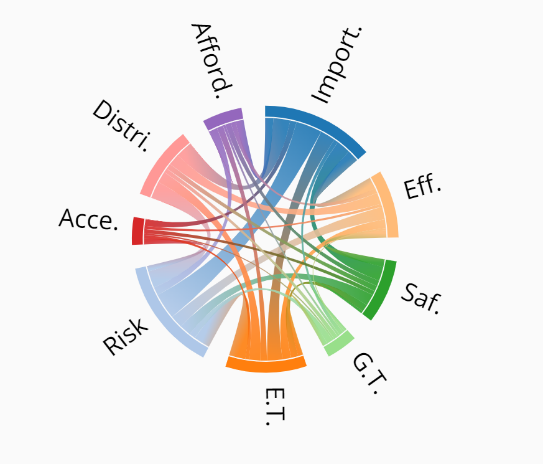 |

**Alternative media:**

| 2020 | 2021 | 2022 |
| --- | --- | --- |
| 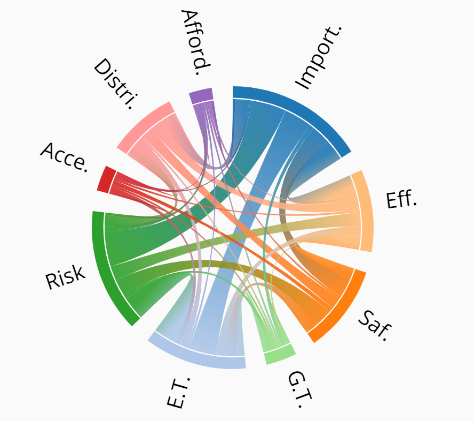 | 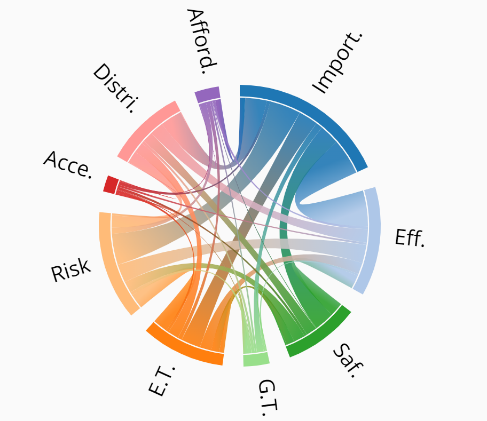 | 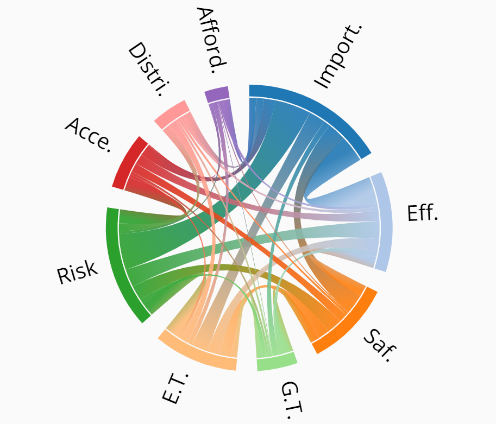 |

**Organization:**

| 2020 | 2021 | 2022 |
| --- | --- | --- |
| 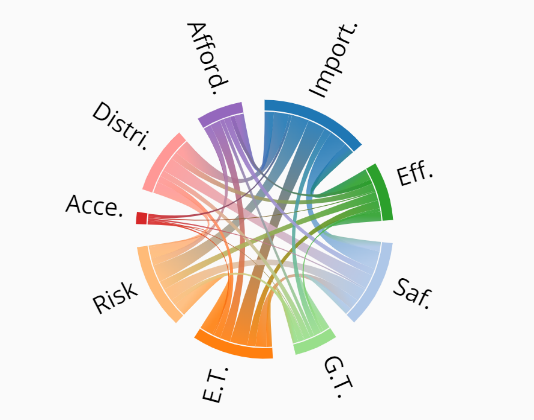 | 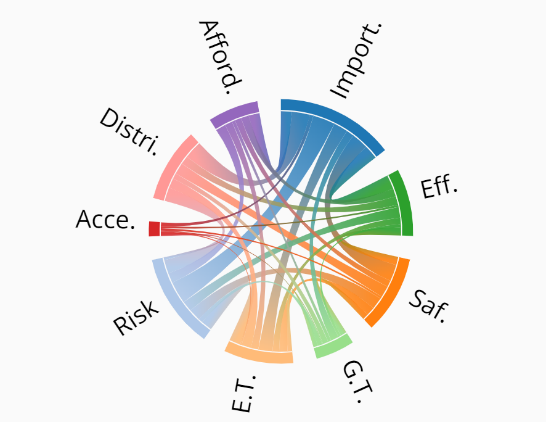 | 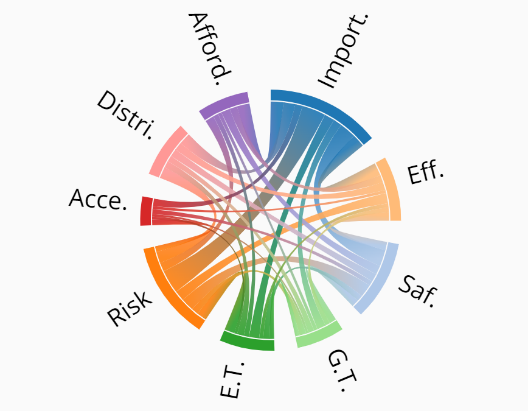 |
| Note: Import. = Vaccine Importance; Eff. = Vaccine Effectiveness; Saf. = Vaccine Safety; G.T. = Government Trust; E.T. = Expert Trust; Acce. = Vaccine Accessibility; Distri. = Vaccine Distribution; Afford. = Vaccine Affordability | | |

**Regular user:**
